# Daily dynamics of ground-dwelling invertebrate communities during and following an extreme high-temperature event in summer 2022, China

**DOI:** 10.1371/journal.pone.0306823

**Published:** 2024-08-23

**Authors:** Meixiang Gao, Jiahuan Sun, Ye Zheng, Tingyu Lu, Jinwen Liu

**Affiliations:** 1 Department of Geography and Spatial Information Techniques, Ningbo University, Ningbo, China; 2 Ningbo Universities Collaborative Innovation Center for Land and Marine Spatial Utilization and Governance Research at Ningbo University, Ningbo, China; 3 Faculty of Electrical Engineering and Computer Science, Ningbo University, Ningbo, China; 4 College of Geography and Environmental Sciences, Hainan Normal University, Haikou, China; 5 Institute of Plant Protection, Jilin Academy of Agricultural Sciences, Changchun, China; Feroze Gandhi Degree College, INDIA

## Abstract

The recent increase in the frequency of extreme weather events and declining soil biodiversity in global agricultural ecosystems make it crucial to assess the daily dynamics of soil communities in fields. To elucidate the daily dynamics of ground-dwelling invertebrate communities, their communities were monitored temporally using infrared camera traps in field farmland during and following an extremely high-temperature (EHT) event in summer 2022 in Ningbo City, China. Nine taxa and 1,147 individuals of the ground-dwelling invertebrate community were photographed in the 176,256 images. There were no significant differences in the taxonomic richness and abundance of the total ground-dwelling invertebrate communities during and following the EHT event. The abundance of ants was significantly decreased following the EHT event, whereas the abundance of other taxa was not. Significantly daily dynamics and obvious differences between each day in taxonomic richness, abundance of ground-dwelling invertebrate community, and abundance of each taxon were not observed during and following the EHT event. The results of this study showed that the daily dynamics of richness and abundance of the ground-dwelling invertebrate community and the abundance of each taxon were not significant during and following the EHT event. Overall, this study provides a useful monitoring method to observe the daily dynamics of ground-dwelling invertebrates in field farmlands and suggests that the daily dynamics of soil fauna communities should be further studied when assessing the effects of climate change on soil biodiversity.

## Introduction

The frequency, duration, and extent of extreme weather events can increase in different regions owing to regional variations [1–3]. For certain organisms that are sensitive to temperature, their metabolism can influence the rates of biochemical reactions [4], and thus, species, especially invertebrates, generally have a limited capacity to adapt to changes at their upper thermal limits [5]. Therefore, extremely high temperatures influence the activity, reproduction, and survival of ground-dwelling invertebrates [6,7]. Understanding the responses of ground-dwelling invertebrates to extremely high temperatures is crucial for protecting the biodiversity of agricultural ecosystems.

Ground-dwelling invertebrates are crucial components of soil faunal communities in agricultural ecosystems. They play important roles in agricultural ecosystem functions, such as supporting crop growth, accelerating nutrient cycling, and improving agricultural ecosystem productivity [8,9]. Soil invertebrates are sensitive to extremely high temperatures with regards to species composition, abundance, species diversity, activity, and distribution [10,11]. For example, when certain soil mesofauna [6] were removed under extremely high-temperature (EHT) conditions in a manipulated experiment, the number of enchytraeids significantly decreased under high temperatures [12] and some species moved deeper into the soil to avoid high-temperature stress [13]. However, ground-dwelling invertebrates are primarily active on the soil surface and are more likely to be affected by extremely high temperatures than soil fauna living in deep soil layers. Although extremely high temperatures can significantly affect the composition of ground-dwelling invertebrates in farmlands [11], whether the daily dynamics of ground-dwelling invertebrates are affected under EHT conditions remains unclear.

Evaluating the daily dynamics of ground-dwelling invertebrates is fundamental to understanding their basic ecology and responses to climate change [14]. Previous studies have focused on the daily dynamics of soil invertebrates at population and community levels [15–17]. Daily activity is one of the most distinctive temporal dynamics of several ant populations [14]. While daily population changes in bacterial-feeding nematodes can have significant wave-like fluctuations in a microcosm experiment, this is not observed in the total nematode communities [18]. However, few studies have focused on the daily dynamics of ground-dwelling invertebrate communities under EHT conditions, especially in complex field environments.

The EHT events that occurred in 2022 resulted in global widespread crop damage, water shortages, wildfires, decreased biodiversity, regional economic losses, and harm to human health [19–21]. Eastern China, especially provinces in the Yangtze River Basin, reached extremely high temperatures characterized by long-lasting, large-scale, and high-impact events in 2022 [21,22]. The EHT events that occurred in 2022 provided us with a valuable opportunity to understand the daily dynamics of ground-dwelling invertebrates on field farmlands.

Collecting daily data on ground-dwelling invertebrates in field farmland is challenging. Recently, infrared camera traps (ICTs) have been proposed as useful tools for investigating the daily dynamics of invertebrates in the field [23,24] and have been successfully utilized to reveal the taxonomic richness and abundance of ground-dwelling invertebrates in farmlands [25]. Therefore, in this study, we collected daily data on ground-dwelling invertebrates using ICTs on a subtropical farmland in Ningbo City, southeastern China, during and following the EHT event in summer 2022. The objective of this study was to reveal (1) the taxonomic richness, abundance of the total ground-dwelling invertebrate community, and the abundance of each taxon during and following the EHT event; and (2) the daily dynamics in taxonomic richness, abundance of the total ground-dwelling invertebrate community, and abundance of each taxon during and following the EHT event. The results of this study shed light on the diversity of ground-dwelling invertebrate communities and their maintenance mechanisms in farmland fields under EHT conditions.

## Materials and methods

### Study site

The study was performed in Ningbo City (28°51′–30°33′ N, 120°55′–122°16′ E), a coastal city located along the eastern coast of China. The altitudes in urban and suburban areas are 4.0–5.8 and 3.6–4.0 m, respectively. The primary soil type is red soil (Argi-Udic Ferrosols under the Chinese Soil Taxonomy and Adults under the United States Department of Agriculture soil classification system) [26]. The climate type is subtropical monsoon, with an average annual temperature and precipitation of 17.4°C and 1480 mm, respectively [27,28].

**Fig 1:**
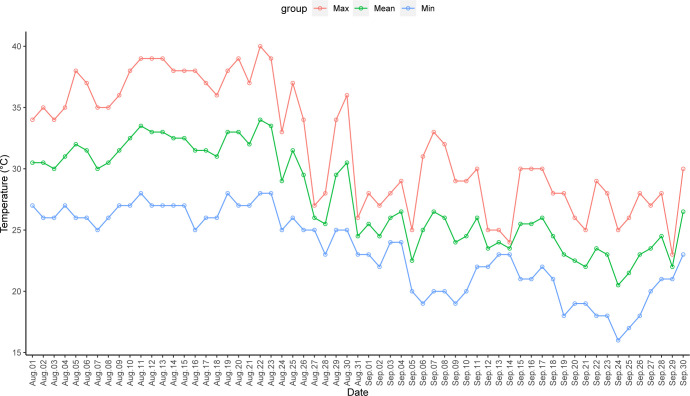
Maximum, minimum, and mean air temperatures in Ningbo from August 1 to September 30, 2022.

A large-scale and persistent EHT event occurred in Ningbo in 2022; with highest temperatures recorded since the meteorological record started in 1953. According to the records of meteorological monitoring stations from different districts of Ningbo, the average number of days with high temperature above 35°C was 50, and the average number of days with high temperature above 38°C is 22.1. These numbers were 29.8 days and 18.7 days longer than in average years, respectively. The most extremely maximum temperatures were above 40°C, which lasted for 4 days [29]. Overall, Ningbo experienced three periods of the EHT event in 2022: July 11–18, July 22–29, and August 5–23 (Fig 1) [29]. Additional information on this event has been described previously [11,28].

### Installation of ICTs and data collection

The experiment was conducted in a Tiansheng Farm vineyard (29°80′ N, 121°40′ E) and was permitted by the managers of the Tiansheng Farm. The vineyards were planted in steel frame greenhouses and covered with plastic throughout the year. Six plots were used in this experiment, each of which was 234 m^2^ and at least 16 m apart (at least including one 1-m-wide canal). One ICT (BG636-48M; Boly Media Communications, Santa Clara, CA, USA) was fixed at the center of each plot, with six ICTs used in total. The camera lens was positioned parallel to and 40 cm above the soil surface. The trigger mode of ICTs was set as a time trigger, which activates at the pre-defined time interval regardless of the specific animal’s motion detection. In this study, each ICT captured three consecutive pictures at 5-min intervals. In other words, three consecutive pictures were taken whenever an ICT was triggered. A quadrat (20 × 20 cm) was placed under one ICT in each plot, with the center of the square facing the ICT lens. Invertebrates that entered the quadrats were photographed by each ICT. The ICTs continuously captured photographs of the plots from 20:00 on July 31 to 24:00 on September 28, 2022. The six ICTs were significantly affected by strong winds and storms caused by typhoons during this experiment. Therefore, certain photographs and records during the typhoons could not be used and were removed from subsequent analysis. To reveal the ground-dwelling invertebrate communities during and following the EHT event, records from August 5 to 22 were used as the period during the EHT event, and records from September 11 to 28 were used as the period following the EHT event. Therefore, data over 34 days (17 days in each of the two periods) and a total of 176,256 images were used in the subsequent analyses. The average maximum, minimum, and mean air temperatures during the EHT event in this experiment were 37.59°C, 26.76°C, and 32.18°C, respectively. The average maximum, minimum, and mean air temperatures following the EHT event were 27.41°C, 20°C, and 23.71°C, respectively. Detailed information of ICTs setting and data collection has been provided in earlier [25,30].

All captured invertebrates were classified into different taxa according to a flowchart for identifying ground-dwelling invertebrates using ICTs [25], as well as information from a previous publication [31]. These invertebrates were identified to the order or family levels. More information about identification and counting of ground-dwelling invertebrates photographed in pictures by ICTs has been provided earlier [25,30].

### Data analysis

Taxonomic richness (number of taxa) and abundance (number of individuals) were determined to evaluate the diversity of ground-dwelling invertebrate communities. Collected data were pooled into 24-h intervals. For clarity, total ground-dwelling invertebrate community refers to the pooled invertebrates during and following the EHT event. The total number of ground-dwelling invertebrate community represents the total number of individuals within the total ground-dwelling invertebrate community.

R is one of the most powerful programming languages for conducting data analysis, modeling, and visualization in community ecology and biodiversity research, therefore, all analysis were conducted using R software (v4.3.1) in this study [32–34]. The distribution of the total ground-dwelling invertebrate communities and each taxon were abnormal during and following the EHT event, based on the normality test using the “ruskal.test” function in the stats package. Therefore, a nonparametric test was used. To evaluate the differences in taxonomic richness and abundances of the total ground-dwelling invertebrate communities and the abundances of each taxon during and following the EHT event, a Mann–Whitney U test was performed using the “wilcox.test” function in the stats package. To evaluate the differences in taxonomic richness and abundances of the total ground-dwelling invertebrate communities and the abundances of each taxon among the monitoring days during and following the EHT event, a Kruskal–Wallis test was performed using the “ruskal.test” function, followed by appropriate multiple testing corrections for a false discovery rate using the “pairwise.wilcox.test” function in the stats package [35]. Statistical significance was defined as *p* < 0.05.

The “boxplot.stats” function in the grDevices package was used to check outliers during and following the EHT events. The abundances of total ground-dwelling invertebrate communities on August 9 (341 ind.), August 10 (319 ind.), and August 22 (449 ind.) were detected as outliers during the EHT event. These outliers were mainly due to the outliers of ant abundances on the same days, with 328, 317, and 394 individuals. The farmers and managers of the Tiansheng Farm remove all potential ant nests in the vineyard throughout the year. We further confirmed that there were no ant nests in the plots during this experiment after checking the area thoroughly. Therefore, we regarded these records on these days as normal data and common phenomenon in subsequent analysis.

## Results

### Taxonomic richness of the total ground-dwelling invertebrate communities during and following the EHT event

Nine taxa were recorded from the total ground-dwelling invertebrate community during the EHT event—ants, beetles, centipedes, earthworms, grasshoppers, millipedes, slugs, snails, and spiders. However, eight taxa were recorded following the EHT event, with centipedes not recorded (Table 1, S1 and S2 Tables).

**Table 1:** Community composition of the total ground-dwelling invertebrate communities during and following the extremely high-temperature event.

		Ants		Slugs		Spiders		Beetles		Centipedes		Millipedes		Grasshoppers		Snails		Earthworms		Total abundance		Taxonomic richness	
Period	Date	Abundance	Std	Abundance	Std	Abundance	Std	Abundance	Std	Abundance	Std	Abundance	Std	Abundance	Std	Abundance	Std	Abundance	Std	Abundance	Std	Richness	Std
During the EHT event	Aug.5	2	0.816	0	0	0	0	3	1.225	0	0	0	0	0	0	0	0	0	0	5	1.329	2	0.516
	Aug.6	6	1.095	0	0	1	0.408	0	0	0	0	0	0	0	0	0	0	0	0	7	0.983	2	0.516
	Aug.7	13	2.639	0	0	0	0	0	0	1	0.408	0	0	0	0	0	0	0	0	14	2.582	2	0.753
	Aug.8	5	1.602	0	0	0	0	1	0.408	0	0	0	0	0	0	0	0	0	0	6	2	2	0.837
	Aug.9	328	124.145	0	0	4	0.516	0	0	2	0.816	2	0.516	3	1.225	2	0.816	0	0	341	125.568	6	1.506
	Aug.10	317	107.654	0	0	0	0	0	0	0	0	2	0.816	0	0	0	0	0	0	319	107.483	2	0.632
	Aug.11	48	13.928	0	0	0	0	3	1.225	0	0	0	0	0	0	0	0	0	0	48	13.928	2	0.632
	Aug.12	14	3.204	0	0	0	0	0	0	0	0	0	0	0	0	0	0	0	0	14	3.204	1	0.548
	Aug.13	12	2.28	0	0	0	0	0	0	0	0	0	0	0	0	0	0	0	0	12	2.28	1	0.548
	Aug.14	23	5.947	0	0	0	0	0	0	0	0	3	1.225	0	0	0	0	0	0	24	6.229	2	0.837
	Aug.15	6	1.673	0	0	0	0	0	0	0	0	0	0	0	0	0	0	0	0	6	1.673	1	0.516
	Aug.17	39	9.094	0	0	0	0	0	0	0	0	0	0	0	0	2	0.816	0	0	41	8.841	2	0.408
	Aug.18	142	17.386	2	0.816	0	0	3	0.837	0	0	12	4.899	3	1.225	3	1.225	0	0	159	21.427	6	1.722
	Aug.19	99	15.984	0	0	4	1.211	0	0	0	0	3	0.837	0	0	1	0.408	1	0.408	109	16.266	5	1.169
	Aug.20	55	11.822	0	0	2	0.816	1	0.408	0	0	13	3.488	1	0.408	0	0	1	0.408	71	11.268	6	1.472
	Aug.21	40	4.59	0	0	0	0	0	0	0	0	10	4.082	1	0.408	0	0	0	0	51	6.411	3	0.753
	Aug.22	394	154.989	0	0	0	0	7	2.858	3	1.225	1	0.408	0	0	49	20.004	0	0	449	177.44	5	1.862
	Abundance	1543		2		11		18		6		46		8		57		2		1676			
Following the EHT event	Sep.11	0	0	0	0	0	0	0	0	0	0	0	0	0	0	0	0	0	0	0	0	0	0
	Sep.12	17	6.94	0	0	0	0	0	0	0	0	2	0.816	0	0	0	0	0	0	19	6.824	2	0.516
	Sep.13	4	1.033	0	0	0	0	0	0	0	0	8	3.266	0	0	0	0	0	0	12	4	2	0.837
	Sep.14	28	9.973	0	0	0	0	0	0	0	0	17	6.94	0	0	0	0	0	0	45	11.077	2	0.753
	Sep.15	15	4.68	0	0	0	0	0	0	0	0	4	1.211	0	0	0	0	0	0	19	4.579	2	0.894
	Sep.16	10	2.582	0	0	0	0	1	0.408	0	0	11	2.563	0	0	0	0	0	0	22	4.803	3	1.265
	Sep.17	6	2	0	0	0	0	0	0	0	0	0	0	0	0	0	0	0	0	6	2	1	0.516
	Sep.18	2	0.816	0	0	0	0	0	0	0	0	0	0	0	0	0	0	0	0	2	0.816	1	0.408
	Sep.19	2	0.816	0	0	0	0	0	0	0	0	0	0	0	0	0	0	0	0	2	0.816	1	0.408
	Sep.21	22	7.202	1	0.408	5	1.329	0	0	0	0	14	4.32	0	0	0	0	0	0	42	11.045	4	1.033
	Sep.22	17	3.71	0	0	15	2.074	0	0	0	0	56	16.17	0	0	0	0	1	0.408	88	15.807	4	0.837
	Sep.23	29	10.381	0	0	19	3.488	0	0	0	0	35	7.627	1	0.408	0	0	0	0	84	13.943	4	0.983
	Sep.24	8	2.805	2	0.816	7	1.602	0	0	0	0	15	4.722	0	0	26	10.614	0	0	58	10.386	5	1.033
	Sep.25	6	1.265	1	0.408	3	1.225	0	0	0	0	5	1.602	0	0	0	0	0	0	15	2.588	4	0.983
	Sep.26	3	0.837	0	0	2	0.816	11	4.491	0	0	0	0	0	0	0	0	0	0	16	4.367	3	0.816
	Sep.27	0	0	0	0	0	0	0	0	0	0	0	0	0	0	0	0	0	0	0	0	0	0
	Sep.28	0	0	0		0	0	0	0	0	0	0	0	1	0.408	0	0	0	0	1	0.408	1	0.408
	Abundance	169		4		51		12		0		167		2		26		1		431			

**Fig 2:**
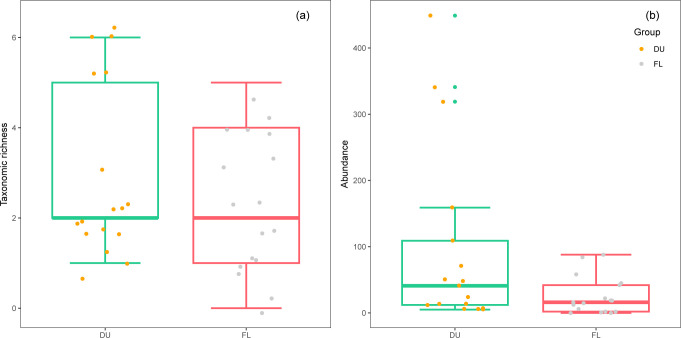
Taxonomic richness and abundance of the total ground-dwelling invertebrate communities during (DU) and following (FL) the extremely high-temperature event.

No significant differences were observed in the taxonomic richness of the total ground-dwelling invertebrate communities between the period during the EHT event and the period following the EHT event (Fig 2A).

**Fig 3:**
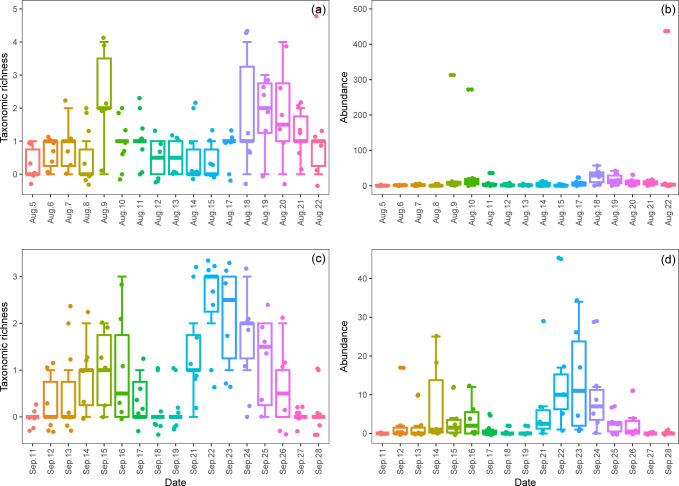
Daily dynamics in taxonomic richness and abundance of the total ground-dwelling invertebrate communities during and following the extremely high-temperature event. (a) and (b) represent taxonomic richness and abundance during the EHT event; (c) and (d) represent taxonomic richness and abundance following the EHT event.

Although there were significant differences in the taxonomic richness of the total ground-dwelling invertebrate communities among the monitoring days during and following the EHT event, respectively, the differences between specific dates did not reach a significant level (Fig 3A and 3C).

### Abundance of the total ground-dwelling invertebrate communities during and following the EHT event

A total of 1,676 individuals were captured from the total ground-dwelling invertebrate community during the EHT event. However, in all, 431 individuals were captured from the total ground-dwelling invertebrate community following the EHT event (Table 1, S1 and S2 Tables).

No significant differences were observed in the abundance of the total ground-dwelling invertebrate communities between the period during and following the EHT event (Fig 2B).

Although there were significant differences in the abundances of the total ground-dwelling invertebrate communities among the monitoring days during and following the EHT event, the differences between specific dates had not reached a significant level (Fig 3B and 3D).

### Abundance of each taxon during and following the EHT event

**Fig 4:**
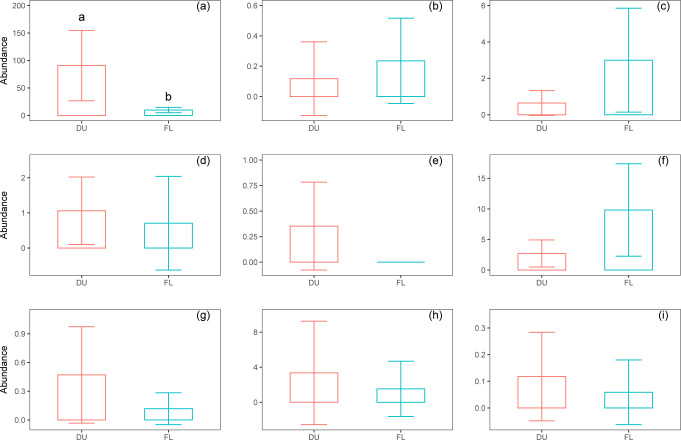
Abundances of nine taxa during (DU) and following (FL) the extremely high-temperature event. (a) Ants; (b) Slugs; (c) Spiders; (d) Beetles; (e) Centipedes; (f) Millipedes; (g) Grasshoppers; (h) Snails; (i) Earthworms.

Ants were the dominant taxon in abundance in both periods, representing 92.06% (1543 ind.) and 39.21% (169 ind.) of the total captures during and following the EHT event, respectively (Table 1). The abundance of ants during the EHT event was significantly higher than that following the EHT event (Fig 4A).

Abundances of beetles (Fig 4D), centipedes (Fig 4E), earthworms (Fig 4I), grasshoppers (Fig 4G), and snails (Fig 4H) during the EHT event were higher than those following the EHT event, although not significantly. Abundances of millipedes (Fig 4F), slugs (Fig 4B), and spiders (Fig 4C) during the EHT event were lower than those following the EHT event, but had not reached a significant level.

**Fig 5:**
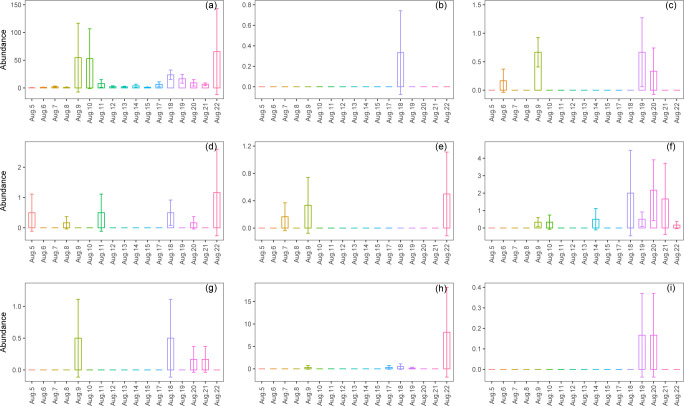
Daily dynamics in abundances of nine taxa during the extremely high-temperature event. (a) Ants; (b) Slugs; (c) Spiders; (d) Beetles; (e) Centipedes; (f) Millipedes; (g) Grasshoppers; (h) Snails; (i) Earthworms.

**Fig 6:**
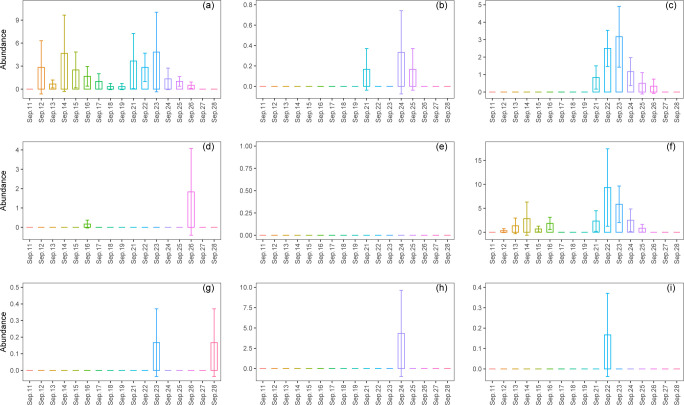
Daily dynamics in abundances of nine taxa following the extremely high-temperature event. (a) Ants; (b) Slugs; (c) Spiders; (d) Beetles; (e) Centipedes; (f) Millipedes; (g) Grasshoppers; (h) Snails; (i) Earthworms.

Although there were significant differences in the abundances of ants and spiders among the monitoring days during the EHT event, these differences between specific dates had not reached a significant level (Fig 5A and 5C). Although there were significant differences in the abundances of spiders and millipedes among the monitoring days following the EHT event, these differences between specific dates had not reached a significant level (Fig 6C and 6F). For other taxa, no significant differences were observed among the monitoring days during and following the EHT event (Figs 5 and 6).

## Discussion

### Daily dynamics of the taxonomic richness and abundance of the total ground-dwelling invertebrate communities during and following EHT event

Though studies mostly reported that extremely high temperatures have negative impacts on the species richness, abundance, biomass, colony success, and reproduction of invertebrates [36–38], higher values in taxonomic richness and abundance of the total ground-dwelling invertebrate community in the farmland were detected during the EHT event in the present study. Although not significant, following the EHT event, a decrease was seen in both taxonomic richness and abundance of the total ground-dwelling invertebrate community. Related studies reported that certain invertebrates could quickly recover from the negative impacts of EHT events, whereas others failed to, depending on their taxa. For example, beetles (*Tribolium castaneum*) can recover from damage to reproductive function within 15 to 28 days following the end of exposure to thermal stress [39]. Certain invertebrates exhibit compensatory mechanisms to mitigate negative impacts from extremely climatic events, but some populations have not recovered from the detrimental effects [40]. However, locusts (*Locusta migratoria*) were unable to recover as normal and their mobilities were limited although temperature decreased to normal after the extremely high temperatures [41]. In the present study, taxonomic richness and abundance of ground-dwelling invertebrate community could not recover 19 days after the EHT event, but endured an insignificant decreasing trend, which was consistent with the soil macrofauna community in the Tiansheng Farm [11]. These results further suggest that the taxonomic richness and abundance of the ground-dwelling invertebrates might experience a negative legacy effect from the EHT event in farmland [11,42].

Taxonomic richness and abundance of the total ground-dwelling invertebrate communities did not show significant daily variations in the farmland over the 17 days during and following the EHT event in the present study. Furthermore, no significant differences in taxonomic richness and abundance of the total ground-dwelling invertebrate communities were observed between certain days during and following the EHT event in the farmland. According to previous studies, the daily dynamics of soil communities exhibited different temporal changes [18,43]. The richness and abundance of the total ground-dwelling beetle communities showed daily temporal dynamics over a continuous 83-day period in a *Pinus koraiensis* plantation although not statistically significant [43], which was consistent with the results of the present study. However, the abundance of the ground-dwelling beetle communities was significantly higher on certain days than other during monitoring period [43]. Significant daily wave-like fluctuations were observed at the population level but not at the community level for bacterial-feeding nematodes in microcosm experiments [18]. Daily changes in the community structure of dung beetles have been observed over 18 days in grasslands, open woodlands, and thickets [44]. Similarly, the daily dynamics of the soil microbial community structure exhibited an oscillatory pattern in a microfield experiment [45]. However, we did not detect significant differences in the richness and abundance of the total ground-dwelling invertebrate communities between specific dates, suggesting an insignificant daily dynamics of these communities in the farmland field, especially under the impacts of extremely high temperatures.

### Daily dynamics of abundances of different taxa during and following the EHT event

Invertebrates response to extremely high temperatures are dynamic and complex, indicating the intricate relationship between these small organisms and their environments [46]. Here, we found that the abundances of ground-dwelling invertebrate taxa corresponding to periods during and after the EHT event were taxonomically dependent. Ground-dwelling ant abundance significantly decreased following the EHT event in the present study. This finding was consistent with another study for soil ants in a facility farmland in Tiansheng Farm, which detected significant decrease in soil ant abundance 18 days following the EHT event in summer 2022 [11]. Abundance of ground-dwelling centipede insignificantly decreased following the EHT event in this study. However, abundance of soil centipede significantly decrease 18 days after the EHT event in a farmland [11]. Abundances of other taxa did not exhibit significant differences for the period following the EHT event compared to the period during the EHT event.

In the present study, there were no significant differences in the abundances of each taxon among the monitoring days during and following the EHT event, and there were no significant differences between specific dates for abundance of each taxon. Significantly daily dynamics of ants [47] and spiders [48,49] were reported in their abundance and activities. However, abundances of ants and spiders did not exhibit significant daily dynamics in both periods during and after the EHT event. Previous studies have reported the daily, seasonal, and/or yearly dynamics of beetles [44,50], centipedes [51,52], grasshoppers [53,54], millipedes [55], snails [56], slugs [57], and earthworms [58]. However, the abundance of these taxa of invertebrates did not show significant variations in the daily changes in the present study.

Results of this study cannot to confirm whether the EHT events considerably impacted the taxonomic richness and abundance of ground-dwelling invertebrate community in farmland as the corresponding records before the EHT event in the same year or during the same months in the previous years were absent. However, monitoring the daily dynamics using ICTs help us to understand the fine-temporal dynamics of ground-dwelling invertebrate community under EHT conditions.

## Conclusions

Using ICTs to collect images from field farmland, nine taxa and 1,147 individuals of ground-dwelling invertebrate communities were monitored during and after an EHT event in Ningbo City, southeastern China. No significant variations in taxonomic richness and abundances of ground-dwelling invertebrate communities were detected during and after the EHT event. Abundance of ant significantly decreased following the EHT event, whereas abundances of other taxa were not significantly altered after the EHT event. Significantly daily dynamics and obvious differences between certain days were not observed for taxonomic richness and abundance of ground-dwelling invertebrate community and abundance of each taxon during and following the EHT event, respectively. This study provides useful and automatic monitoring methods (ICTs) to monitor the daily dynamics of ground-dwelling invertebrate communities under extremely high temperature conditions in field farmlands. These findings contribute to informed decision-making regarding biodiversity protection and farmland management strategies.

## Supporting information

S1 Table Daily community composition of ground-dwelling invertebrate communities in each plot, monitored using infrared camera traps (ICTs), during the extremely high-temperature event (DU). (DOCX)

S2 Table Daily community composition of ground-dwelling invertebrate communities in each plot, monitored using infrared camera traps (ICTs), following the extremely high-temperature event (FL). (DOCX)
