# Supplementary material for: Daily dynamics of ground-dwelling invertebrate communities during and following an extreme high-temperature event in summer 2022, China
Source: PLoS One. 2024 Aug 23;19(8):e0306823. doi: 10.1371/journal.pone.0306823 (PMC11343418; doi:10.1371/journal.pone.0306823)
Supplement: S1 Table — (DOCX) [file pone.0306823.s001.docx]

**Table S1** Daily community composition of ground-dwelling invertebrate communities in each plot, monitored using infrared camera traps (ICTs), during the extremely high-temperature event (DU).

| Period | Camera | Date | Ants | Slugs | Spiders | Beetles | Centipedes | Millipedes | Grasshoppers | Snails | Earthworms | Abundance | Richness |
| --- | --- | --- | --- | --- | --- | --- | --- | --- | --- | --- | --- | --- | --- |
| DU | C2 | Aug.5 | 0 | 0 | 0 | 0 | 0 | 0 | 0 | 0 | 0 | 0 | 0 |
| DU | C3 | Aug.5 | 0 | 0 | 0 | 0 | 0 | 0 | 0 | 0 | 0 | 0 | 0 |
| DU | C4 | Aug.5 | 0 | 0 | 0 | 0 | 0 | 0 | 0 | 0 | 0 | 0 | 0 |
| DU | C6 | Aug.5 | 0 | 0 | 0 | 0 | 0 | 0 | 0 | 0 | 0 | 0 | 0 |
| DU | C8 | Aug.5 | 0 | 0 | 0 | 3 | 0 | 0 | 0 | 0 | 0 | 3 | 1 |
| DU | C10 | Aug.5 | 2 | 0 | 0 | 0 | 0 | 0 | 0 | 0 | 0 | 2 | 1 |
| DU | C2 | Aug.6 | 2 | 0 | 0 | 0 | 0 | 0 | 0 | 0 | 0 | 2 | 1 |
| DU | C3 | Aug.6 | 0 | 0 | 0 | 0 | 0 | 0 | 0 | 0 | 0 | 0 | 0 |
| DU | C4 | Aug.6 | 0 | 0 | 1 | 0 | 0 | 0 | 0 | 0 | 0 | 1 | 1 |
| DU | C6 | Aug.6 | 0 | 0 | 0 | 0 | 0 | 0 | 0 | 0 | 0 | 0 | 0 |
| DU | C8 | Aug.6 | 2 | 0 | 0 | 0 | 0 | 0 | 0 | 0 | 0 | 2 | 1 |
| DU | C10 | Aug.6 | 2 | 0 | 0 | 0 | 0 | 0 | 0 | 0 | 0 | 2 | 1 |
| DU | C2 | Aug.7 | 5 | 0 | 0 | 0 | 0 | 0 | 0 | 0 | 0 | 5 | 1 |
| DU | C3 | Aug.7 | 6 | 0 | 0 | 0 | 0 | 0 | 0 | 0 | 0 | 6 | 1 |
| DU | C4 | Aug.7 | 1 | 0 | 0 | 0 | 0 | 0 | 0 | 0 | 0 | 1 | 1 |
| DU | C6 | Aug.7 | 0 | 0 | 0 | 0 | 0 | 0 | 0 | 0 | 0 | 0 | 0 |
| DU | C8 | Aug.7 | 1 | 0 | 0 | 0 | 1 | 0 | 0 | 0 | 0 | 2 | 2 |
| DU | C10 | Aug.7 | 0 | 0 | 0 | 0 | 0 | 0 | 0 | 0 | 0 | 0 | 0 |
| DU | C2 | Aug.8 | 0 | 0 | 0 | 0 | 0 | 0 | 0 | 0 | 0 | 0 | 0 |
| DU | C3 | Aug.8 | 4 | 0 | 0 | 1 | 0 | 0 | 0 | 0 | 0 | 5 | 2 |
| DU | C4 | Aug.8 | 1 | 0 | 0 | 0 | 0 | 0 | 0 | 0 | 0 | 1 | 1 |
| DU | C6 | Aug.8 | 0 | 0 | 0 | 0 | 0 | 0 | 0 | 0 | 0 | 0 | 0 |
| DU | C8 | Aug.8 | 0 | 0 | 0 | 0 | 0 | 0 | 0 | 0 | 0 | 0 | 0 |
| DU | C10 | Aug.8 | 0 | 0 | 0 | 0 | 0 | 0 | 0 | 0 | 0 | 0 | 0 |
| DU | C2 | Aug.9 | 5 | 0 | 1 | 0 | 0 | 0 | 0 | 0 | 0 | 6 | 2 |
| DU | C3 | Aug.9 | 308 | 0 | 1 | 0 | 0 | 1 | 3 | 0 | 0 | 313 | 4 |
| DU | C4 | Aug.9 | 8 | 0 | 1 | 0 | 0 | 1 | 0 | 2 | 0 | 12 | 4 |
| DU | C6 | Aug.9 | 6 | 0 | 0 | 0 | 2 | 0 | 0 | 0 | 0 | 8 | 2 |
| DU | C8 | Aug.9 | 1 | 0 | 1 | 0 | 0 | 0 | 0 | 0 | 0 | 2 | 2 |
| DU | C10 | Aug.9 | 0 | 0 | 0 | 0 | 0 | 0 | 0 | 0 | 0 | 0 | 0 |
| DU | C2 | Aug.10 | 15 | 0 | 0 | 0 | 0 | 0 | 0 | 0 | 0 | 15 | 1 |
| DU | C3 | Aug.10 | 272 | 0 | 0 | 0 | 0 | 0 | 0 | 0 | 0 | 272 | 1 |
| DU | C4 | Aug.10 | 6 | 0 | 0 | 0 | 0 | 2 | 0 | 0 | 0 | 8 | 2 |
| DU | C6 | Aug.10 | 3 | 0 | 0 | 0 | 0 | 0 | 0 | 0 | 0 | 3 | 1 |
| DU | C8 | Aug.10 | 21 | 0 | 0 | 0 | 0 | 0 | 0 | 0 | 0 | 21 | 1 |
| DU | C10 | Aug.10 | 0 | 0 | 0 | 0 | 0 | 0 | 0 | 0 | 0 | 0 | 0 |
| DU | C2 | Aug.11 | 2 | 0 | 0 | 0 | 0 | 0 | 0 | 0 | 0 | 2 | 1 |
| DU | C3 | Aug.11 | 36 | 0 | 0 | 3 | 0 | 0 | 0 | 0 | 0 | 36 | 2 |
| DU | C4 | Aug.11 | 1 | 0 | 0 | 0 | 0 | 0 | 0 | 0 | 0 | 1 | 1 |
| DU | C6 | Aug.11 | 2 | 0 | 0 | 0 | 0 | 0 | 0 | 0 | 0 | 2 | 1 |
| DU | C8 | Aug.11 | 7 | 0 | 0 | 0 | 0 | 0 | 0 | 0 | 0 | 7 | 1 |
| DU | C10 | Aug.11 | 0 | 0 | 0 | 0 | 0 | 0 | 0 | 0 | 0 | 0 | 0 |
| DU | C2 | Aug.12 | 2 | 0 | 0 | 0 | 0 | 0 | 0 | 0 | 0 | 2 | 1 |
| DU | C3 | Aug.12 | 4 | 0 | 0 | 0 | 0 | 0 | 0 | 0 | 0 | 4 | 1 |
| DU | C4 | Aug.12 | 0 | 0 | 0 | 0 | 0 | 0 | 0 | 0 | 0 | 0 | 0 |
| DU | C6 | Aug.12 | 0 | 0 | 0 | 0 | 0 | 0 | 0 | 0 | 0 | 0 | 0 |
| DU | C8 | Aug.12 | 8 | 0 | 0 | 0 | 0 | 0 | 0 | 0 | 0 | 8 | 1 |
| DU | C10 | Aug.12 | 0 | 0 | 0 | 0 | 0 | 0 | 0 | 0 | 0 | 0 | 0 |
| DU | C2 | Aug.13 | 3 | 0 | 0 | 0 | 0 | 0 | 0 | 0 | 0 | 3 | 1 |
| DU | C3 | Aug.13 | 5 | 0 | 0 | 0 | 0 | 0 | 0 | 0 | 0 | 5 | 1 |
| DU | C4 | Aug.13 | 0 | 0 | 0 | 0 | 0 | 0 | 0 | 0 | 0 | 0 | 0 |
| DU | C6 | Aug.13 | 0 | 0 | 0 | 0 | 0 | 0 | 0 | 0 | 0 | 0 | 0 |
| DU | C8 | Aug.13 | 4 | 0 | 0 | 0 | 0 | 0 | 0 | 0 | 0 | 4 | 1 |
| DU | C10 | Aug.13 | 0 | 0 | 0 | 0 | 0 | 0 | 0 | 0 | 0 | 0 | 0 |
| DU | C2 | Aug.14 | 12 | 0 | 0 | 0 | 0 | 3 | 0 | 0 | 0 | 13 | 2 |
| DU | C3 | Aug.14 | 0 | 0 | 0 | 0 | 0 | 0 | 0 | 0 | 0 | 0 | 0 |
| DU | C4 | Aug.14 | 0 | 0 | 0 | 0 | 0 | 0 | 0 | 0 | 0 | 0 | 0 |
| DU | C6 | Aug.14 | 0 | 0 | 0 | 0 | 0 | 0 | 0 | 0 | 0 | 0 | 0 |
| DU | C8 | Aug.14 | 11 | 0 | 0 | 0 | 0 | 0 | 0 | 0 | 0 | 11 | 1 |
| DU | C10 | Aug.14 | 0 | 0 | 0 | 0 | 0 | 0 | 0 | 0 | 0 | 0 | 0 |
| DU | C2 | Aug.15 | 2 | 0 | 0 | 0 | 0 | 0 | 0 | 0 | 0 | 2 | 1 |
| DU | C3 | Aug.15 | 0 | 0 | 0 | 0 | 0 | 0 | 0 | 0 | 0 | 0 | 0 |
| DU | C4 | Aug.15 | 0 | 0 | 0 | 0 | 0 | 0 | 0 | 0 | 0 | 0 | 0 |
| DU | C6 | Aug.15 | 0 | 0 | 0 | 0 | 0 | 0 | 0 | 0 | 0 | 0 | 0 |
| DU | C8 | Aug.15 | 4 | 0 | 0 | 0 | 0 | 0 | 0 | 0 | 0 | 4 | 1 |
| DU | C10 | Aug.15 | 0 | 0 | 0 | 0 | 0 | 0 | 0 | 0 | 0 | 0 | 0 |
| DU | C2 | Aug.17 | 0 | 0 | 0 | 0 | 0 | 0 | 0 | 2 | 0 | 2 | 1 |
| DU | C3 | Aug.17 | 23 | 0 | 0 | 0 | 0 | 0 | 0 | 0 | 0 | 23 | 1 |
| DU | C4 | Aug.17 | 4 | 0 | 0 | 0 | 0 | 0 | 0 | 0 | 0 | 4 | 1 |
| DU | C6 | Aug.17 | 0 | 0 | 0 | 0 | 0 | 0 | 0 | 0 | 0 | 0 | 0 |
| DU | C8 | Aug.17 | 11 | 0 | 0 | 0 | 0 | 0 | 0 | 0 | 0 | 11 | 1 |
| DU | C10 | Aug.17 | 1 | 0 | 0 | 0 | 0 | 0 | 0 | 0 | 0 | 1 | 1 |
| DU | C2 | Aug.18 | 0 | 0 | 0 | 0 | 0 | 0 | 0 | 0 | 0 | 0 | 0 |
| DU | C3 | Aug.18 | 37 | 0 | 0 | 2 | 0 | 0 | 3 | 3 | 0 | 39 | 4 |
| DU | C4 | Aug.18 | 42 | 2 | 0 | 1 | 0 | 12 | 0 | 0 | 0 | 57 | 4 |
| DU | C6 | Aug.18 | 25 | 0 | 0 | 0 | 0 | 0 | 0 | 0 | 0 | 25 | 1 |
| DU | C8 | Aug.18 | 33 | 0 | 0 | 0 | 0 | 0 | 0 | 0 | 0 | 33 | 1 |
| DU | C10 | Aug.18 | 5 | 0 | 0 | 0 | 0 | 0 | 0 | 0 | 0 | 5 | 1 |
| DU | C2 | Aug.19 | 11 | 0 | 1 | 0 | 0 | 1 | 0 | 0 | 0 | 13 | 3 |
| DU | C3 | Aug.19 | 0 | 0 | 0 | 0 | 0 | 0 | 0 | 0 | 0 | 0 | 0 |
| DU | C4 | Aug.19 | 29 | 0 | 0 | 0 | 0 | 2 | 0 | 1 | 0 | 33 | 3 |
| DU | C6 | Aug.19 | 13 | 0 | 3 | 0 | 0 | 0 | 0 | 0 | 0 | 16 | 2 |
| DU | C8 | Aug.19 | 42 | 0 | 0 | 0 | 0 | 0 | 0 | 0 | 0 | 42 | 1 |
| DU | C10 | Aug.19 | 4 | 0 | 0 | 0 | 0 | 0 | 0 | 0 | 1 | 5 | 2 |
| DU | C2 | Aug.20 | 6 | 0 | 2 | 0 | 0 | 5 | 1 | 0 | 0 | 12 | 4 |
| DU | C3 | Aug.20 | 0 | 0 | 0 | 0 | 0 | 0 | 0 | 0 | 0 | 0 | 0 |
| DU | C4 | Aug.20 | 3 | 0 | 0 | 1 | 0 | 8 | 0 | 0 | 0 | 12 | 3 |
| DU | C6 | Aug.20 | 14 | 0 | 0 | 0 | 0 | 0 | 0 | 0 | 1 | 15 | 2 |
| DU | C8 | Aug.20 | 31 | 0 | 0 | 0 | 0 | 0 | 0 | 0 | 0 | 31 | 1 |
| DU | C10 | Aug.20 | 1 | 0 | 0 | 0 | 0 | 0 | 0 | 0 | 0 | 1 | 1 |
| DU | C2 | Aug.21 | 13 | 0 | 0 | 0 | 0 | 0 | 1 | 0 | 0 | 14 | 2 |
| DU | C3 | Aug.21 | 0 | 0 | 0 | 0 | 0 | 0 | 0 | 0 | 0 | 0 | 0 |
| DU | C4 | Aug.21 | 7 | 0 | 0 | 0 | 0 | 10 | 0 | 0 | 0 | 17 | 2 |
| DU | C6 | Aug.21 | 8 | 0 | 0 | 0 | 0 | 0 | 0 | 0 | 0 | 8 | 1 |
| DU | C8 | Aug.21 | 9 | 0 | 0 | 0 | 0 | 0 | 0 | 0 | 0 | 9 | 1 |
| DU | C10 | Aug.21 | 3 | 0 | 0 | 0 | 0 | 0 | 0 | 0 | 0 | 3 | 1 |
| DU | C2 | Aug.22 | 2 | 0 | 0 | 0 | 0 | 0 | 0 | 0 | 0 | 2 | 1 |
| DU | C3 | Aug.22 | 382 | 0 | 0 | 7 | 3 | 1 | 0 | 49 | 0 | 437 | 5 |
| DU | C4 | Aug.22 | 0 | 0 | 0 | 0 | 0 | 0 | 0 | 0 | 0 | 0 | 0 |
| DU | C6 | Aug.22 | 0 | 0 | 0 | 0 | 0 | 0 | 0 | 0 | 0 | 0 | 0 |
| DU | C8 | Aug.22 | 4 | 0 | 0 | 0 | 0 | 0 | 0 | 0 | 0 | 4 | 1 |
| DU | C10 | Aug.22 | 6 | 0 | 0 | 0 | 0 | 0 | 0 | 0 | 0 | 6 | 1 |

Note: DU represents the period during the extremely high-temperature event. C2, C3, C4, C6, C8, and C10 represent the plots that were used in this study.
