# Supplementary material for: Daily dynamics of ground-dwelling invertebrate communities during and following an extreme high-temperature event in summer 2022, China
Source: PLoS One. 2024 Aug 23;19(8):e0306823. doi: 10.1371/journal.pone.0306823 (PMC11343418; doi:10.1371/journal.pone.0306823)
Supplement: S2 Table — (DOCX) [file pone.0306823.s002.docx]

**Table S2** Daily community composition of ground-dwelling invertebrate communities in each plot, monitored using infrared camera traps (ICTs), following the extremely high-temperature event (FL).

| Period | Camera | Date | Ants | Slugs | Spiders | Beetles | Centipedes | Millipedes | Grasshoppers | Snails | Earthworms | Abundance | Richness |
| --- | --- | --- | --- | --- | --- | --- | --- | --- | --- | --- | --- | --- | --- |
| FL | C2 | Sep.11 | 0 | 0 | 0 | 0 | 0 | 0 | 0 | 0 | 0 | 0 | 0 |
| FL | C3 | Sep.11 | 0 | 0 | 0 | 0 | 0 | 0 | 0 | 0 | 0 | 0 | 0 |
| FL | C4 | Sep.11 | 0 | 0 | 0 | 0 | 0 | 0 | 0 | 0 | 0 | 0 | 0 |
| FL | C6 | Sep.11 | 0 | 0 | 0 | 0 | 0 | 0 | 0 | 0 | 0 | 0 | 0 |
| FL | C8 | Sep.11 | 0 | 0 | 0 | 0 | 0 | 0 | 0 | 0 | 0 | 0 | 0 |
| FL | C10 | Sep.11 | 0 | 0 | 0 | 0 | 0 | 0 | 0 | 0 | 0 | 0 | 0 |
| FL | C2 | Sep.12 | 0 | 0 | 0 | 0 | 0 | 2 | 0 | 0 | 0 | 2 | 1 |
| FL | C3 | Sep.12 | 17 | 0 | 0 | 0 | 0 | 0 | 0 | 0 | 0 | 17 | 1 |
| FL | C4 | Sep.12 | 0 | 0 | 0 | 0 | 0 | 0 | 0 | 0 | 0 | 0 | 0 |
| FL | C6 | Sep.12 | 0 | 0 | 0 | 0 | 0 | 0 | 0 | 0 | 0 | 0 | 0 |
| FL | C8 | Sep.12 | 0 | 0 | 0 | 0 | 0 | 0 | 0 | 0 | 0 | 0 | 0 |
| FL | C10 | Sep.12 | 0 | 0 | 0 | 0 | 0 | 0 | 0 | 0 | 0 | 0 | 0 |
| FL | C2 | Sep.13 | 2 | 0 | 0 | 0 | 0 | 8 | 0 | 0 | 0 | 10 | 2 |
| FL | C3 | Sep.13 | 2 | 0 | 0 | 0 | 0 | 0 | 0 | 0 | 0 | 2 | 1 |
| FL | C4 | Sep.13 | 0 | 0 | 0 | 0 | 0 | 0 | 0 | 0 | 0 | 0 | 0 |
| FL | C6 | Sep.13 | 0 | 0 | 0 | 0 | 0 | 0 | 0 | 0 | 0 | 0 | 0 |
| FL | C8 | Sep.13 | 0 | 0 | 0 | 0 | 0 | 0 | 0 | 0 | 0 | 0 | 0 |
| FL | C10 | Sep.13 | 0 | 0 | 0 | 0 | 0 | 0 | 0 | 0 | 0 | 0 | 0 |
| FL | C2 | Sep.14 | 1 | 0 | 0 | 0 | 0 | 17 | 0 | 0 | 0 | 18 | 2 |
| FL | C3 | Sep.14 | 0 | 0 | 0 | 0 | 0 | 0 | 0 | 0 | 0 | 0 | 0 |
| FL | C4 | Sep.14 | 1 | 0 | 0 | 0 | 0 | 0 | 0 | 0 | 0 | 1 | 1 |
| FL | C6 | Sep.14 | 1 | 0 | 0 | 0 | 0 | 0 | 0 | 0 | 0 | 1 | 1 |
| FL | C8 | Sep.14 | 25 | 0 | 0 | 0 | 0 | 0 | 0 | 0 | 0 | 25 | 1 |
| FL | C10 | Sep.14 | 0 | 0 | 0 | 0 | 0 | 0 | 0 | 0 | 0 | 0 | 0 |
| FL | C2 | Sep.15 | 1 | 0 | 0 | 0 | 0 | 3 | 0 | 0 | 0 | 4 | 2 |
| FL | C3 | Sep.15 | 0 | 0 | 0 | 0 | 0 | 0 | 0 | 0 | 0 | 0 | 0 |
| FL | C4 | Sep.15 | 1 | 0 | 0 | 0 | 0 | 1 | 0 | 0 | 0 | 2 | 2 |
| FL | C6 | Sep.15 | 0 | 0 | 0 | 0 | 0 | 0 | 0 | 0 | 0 | 0 | 0 |
| FL | C8 | Sep.15 | 12 | 0 | 0 | 0 | 0 | 0 | 0 | 0 | 0 | 12 | 1 |
| FL | C10 | Sep.15 | 1 | 0 | 0 | 0 | 0 | 0 | 0 | 0 | 0 | 1 | 1 |
| FL | C2 | Sep.16 | 0 | 0 | 0 | 0 | 0 | 4 | 0 | 0 | 0 | 4 | 1 |
| FL | C3 | Sep.16 | 0 | 0 | 0 | 0 | 0 | 0 | 0 | 0 | 0 | 0 | 0 |
| FL | C4 | Sep.16 | 5 | 0 | 0 | 1 | 0 | 6 | 0 | 0 | 0 | 12 | 3 |
| FL | C6 | Sep.16 | 0 | 0 | 0 | 0 | 0 | 0 | 0 | 0 | 0 | 0 | 0 |
| FL | C8 | Sep.16 | 5 | 0 | 0 | 0 | 0 | 1 | 0 | 0 | 0 | 6 | 2 |
| FL | C10 | Sep.16 | 0 | 0 | 0 | 0 | 0 | 0 | 0 | 0 | 0 | 0 | 0 |
| FL | C2 | Sep.17 | 1 | 0 | 0 | 0 | 0 | 0 | 0 | 0 | 0 | 1 | 1 |
| FL | C3 | Sep.17 | 0 | 0 | 0 | 0 | 0 | 0 | 0 | 0 | 0 | 0 | 0 |
| FL | C4 | Sep.17 | 0 | 0 | 0 | 0 | 0 | 0 | 0 | 0 | 0 | 0 | 0 |
| FL | C6 | Sep.17 | 0 | 0 | 0 | 0 | 0 | 0 | 0 | 0 | 0 | 0 | 0 |
| FL | C8 | Sep.17 | 5 | 0 | 0 | 0 | 0 | 0 | 0 | 0 | 0 | 5 | 1 |
| FL | C10 | Sep.17 | 0 | 0 | 0 | 0 | 0 | 0 | 0 | 0 | 0 | 0 | 0 |
| FL | C2 | Sep.18 | 0 | 0 | 0 | 0 | 0 | 0 | 0 | 0 | 0 | 0 | 0 |
| FL | C3 | Sep.18 | 0 | 0 | 0 | 0 | 0 | 0 | 0 | 0 | 0 | 0 | 0 |
| FL | C4 | Sep.18 | 0 | 0 | 0 | 0 | 0 | 0 | 0 | 0 | 0 | 0 | 0 |
| FL | C6 | Sep.18 | 0 | 0 | 0 | 0 | 0 | 0 | 0 | 0 | 0 | 0 | 0 |
| FL | C8 | Sep.18 | 2 | 0 | 0 | 0 | 0 | 0 | 0 | 0 | 0 | 2 | 1 |
| FL | C10 | Sep.18 | 0 | 0 | 0 | 0 | 0 | 0 | 0 | 0 | 0 | 0 | 0 |
| FL | C2 | Sep.19 | 0 | 0 | 0 | 0 | 0 | 0 | 0 | 0 | 0 | 0 | 0 |
| FL | C3 | Sep.19 | 0 | 0 | 0 | 0 | 0 | 0 | 0 | 0 | 0 | 0 | 0 |
| FL | C4 | Sep.19 | 0 | 0 | 0 | 0 | 0 | 0 | 0 | 0 | 0 | 0 | 0 |
| FL | C6 | Sep.19 | 0 | 0 | 0 | 0 | 0 | 0 | 0 | 0 | 0 | 0 | 0 |
| FL | C8 | Sep.19 | 2 | 0 | 0 | 0 | 0 | 0 | 0 | 0 | 0 | 2 | 1 |
| FL | C10 | Sep.19 | 0 | 0 | 0 | 0 | 0 | 0 | 0 | 0 | 0 | 0 | 0 |
| FL | C2 | Sep.21 | 0 | 1 | 0 | 0 | 0 | 0 | 0 | 0 | 0 | 1 | 1 |
| FL | C3 | Sep.21 | 18 | 0 | 0 | 0 | 0 | 11 | 0 | 0 | 0 | 29 | 2 |
| FL | C4 | Sep.21 | 0 | 0 | 0 | 0 | 0 | 0 | 0 | 0 | 0 | 0 | 0 |
| FL | C6 | Sep.21 | 0 | 0 | 3 | 0 | 0 | 0 | 0 | 0 | 0 | 3 | 1 |
| FL | C8 | Sep.21 | 4 | 0 | 2 | 0 | 0 | 1 | 0 | 0 | 0 | 7 | 3 |
| FL | C10 | Sep.21 | 0 | 0 | 0 | 0 | 0 | 2 | 0 | 0 | 0 | 2 | 1 |
| FL | C2 | Sep.22 | 2 | 0 | 3 | 0 | 0 | 5 | 0 | 0 | 0 | 10 | 3 |
| FL | C3 | Sep.22 | 2 | 0 | 2 | 0 | 0 | 42 | 0 | 0 | 0 | 45 | 3 |
| FL | C4 | Sep.22 | 0 | 0 | 3 | 0 | 0 | 2 | 0 | 0 | 0 | 5 | 2 |
| FL | C6 | Sep.22 | 3 | 0 | 1 | 0 | 0 | 6 | 0 | 0 | 0 | 10 | 3 |
| FL | C8 | Sep.22 | 10 | 0 | 6 | 0 | 0 | 1 | 0 | 0 | 0 | 17 | 3 |
| FL | C10 | Sep.22 | 0 | 0 | 0 | 0 | 0 | 0 | 0 | 0 | 1 | 1 | 1 |
| FL | C2 | Sep.23 | 0 | 0 | 2 | 0 | 0 | 3 | 0 | 0 | 0 | 5 | 2 |
| FL | C3 | Sep.23 | 1 | 0 | 2 | 0 | 0 | 14 | 0 | 0 | 0 | 17 | 3 |
| FL | C4 | Sep.23 | 1 | 0 | 8 | 0 | 0 | 17 | 0 | 0 | 0 | 26 | 3 |
| FL | C6 | Sep.23 | 1 | 0 | 0 | 0 | 0 | 0 | 0 | 0 | 0 | 1 | 1 |
| FL | C8 | Sep.23 | 26 | 0 | 7 | 0 | 0 | 0 | 1 | 0 | 0 | 34 | 3 |
| FL | C10 | Sep.23 | 0 | 0 | 0 | 0 | 0 | 1 | 0 | 0 | 0 | 1 | 1 |
| FL | C2 | Sep.24 | 0 | 2 | 1 | 0 | 0 | 0 | 0 | 26 | 0 | 29 | 3 |
| FL | C3 | Sep.24 | 0 | 0 | 0 | 0 | 0 | 12 | 0 | 0 | 0 | 12 | 1 |
| FL | C4 | Sep.24 | 0 | 0 | 4 | 0 | 0 | 1 | 0 | 0 | 0 | 5 | 2 |
| FL | C6 | Sep.24 | 1 | 0 | 0 | 0 | 0 | 2 | 0 | 0 | 0 | 3 | 2 |
| FL | C8 | Sep.24 | 7 | 0 | 2 | 0 | 0 | 0 | 0 | 0 | 0 | 9 | 2 |
| FL | C10 | Sep.24 | 0 | 0 | 0 | 0 | 0 | 0 | 0 | 0 | 0 | 0 | 0 |
| FL | C2 | Sep.25 | 1 | 1 | 0 | 0 | 0 | 0 | 0 | 0 | 0 | 2 | 2 |
| FL | C3 | Sep.25 | 0 | 0 | 0 | 0 | 0 | 0 | 0 | 0 | 0 | 0 | 0 |
| FL | C4 | Sep.25 | 0 | 0 | 3 | 0 | 0 | 4 | 0 | 0 | 0 | 7 | 2 |
| FL | C6 | Sep.25 | 0 | 0 | 0 | 0 | 0 | 0 | 0 | 0 | 0 | 0 | 0 |
| FL | C8 | Sep.25 | 3 | 0 | 0 | 0 | 0 | 0 | 0 | 0 | 0 | 3 | 1 |
| FL | C10 | Sep.25 | 2 | 0 | 0 | 0 | 0 | 1 | 0 | 0 | 0 | 3 | 2 |
| FL | C2 | Sep.26 | 2 | 0 | 2 | 0 | 0 | 0 | 0 | 0 | 0 | 4 | 2 |
| FL | C3 | Sep.26 | 0 | 0 | 0 | 0 | 0 | 0 | 0 | 0 | 0 | 0 | 0 |
| FL | C4 | Sep.26 | 0 | 0 | 0 | 0 | 0 | 0 | 0 | 0 | 0 | 0 | 0 |
| FL | C6 | Sep.26 | 0 | 0 | 0 | 0 | 0 | 0 | 0 | 0 | 0 | 0 | 0 |
| FL | C8 | Sep.26 | 1 | 0 | 0 | 0 | 0 | 0 | 0 | 0 | 0 | 1 | 1 |
| FL | C10 | Sep.26 | 0 | 0 | 0 | 11 | 0 | 0 | 0 | 0 | 0 | 11 | 1 |
| FL | C2 | Sep.27 | 0 | 0 | 0 | 0 | 0 | 0 | 0 | 0 | 0 | 0 | 0 |
| FL | C3 | Sep.27 | 0 | 0 | 0 | 0 | 0 | 0 | 0 | 0 | 0 | 0 | 0 |
| FL | C4 | Sep.27 | 0 | 0 | 0 | 0 | 0 | 0 | 0 | 0 | 0 | 0 | 0 |
| FL | C6 | Sep.27 | 0 | 0 | 0 | 0 | 0 | 0 | 0 | 0 | 0 | 0 | 0 |
| FL | C8 | Sep.27 | 0 | 0 | 0 | 0 | 0 | 0 | 0 | 0 | 0 | 0 | 0 |
| FL | C10 | Sep.27 | 0 | 0 | 0 | 0 | 0 | 0 | 0 | 0 | 0 | 0 | 0 |
| FL | C2 | Sep.28 | 0 | 0 | 0 | 0 | 0 | 0 | 0 | 0 | 0 | 0 | 0 |
| FL | C3 | Sep.28 | 0 | 0 | 0 | 0 | 0 | 0 | 0 | 0 | 0 | 0 | 0 |
| FL | C4 | Sep.28 | 0 | 0 | 0 | 0 | 0 | 0 | 0 | 0 | 0 | 0 | 0 |
| FL | C6 | Sep.28 | 0 | 0 | 0 | 0 | 0 | 0 | 1 | 0 | 0 | 1 | 1 |
| FL | C8 | Sep.28 | 0 | 0 | 0 | 0 | 0 | 0 | 0 | 0 | 0 | 0 | 0 |
| FL | C10 | Sep.28 | 0 | 0 | 0 | 0 | 0 | 0 | 0 | 0 | 0 | 0 | 0 |

Note: FL represents the period following the extremely high-temperature event. C2, C3, C4, C6, C8, and C10 represent the plots that were used in this study.
